# Validation of a reference interval for symmetric dimethylarginine in healthy goats and its comparison to values in goats with obstructive urolithiasis

**DOI:** 10.1111/jvim.17162

**Published:** 2024-08-17

**Authors:** Blanca E. Camacho, Siena L. Mitman, Derek M. Foster, Jennifer Halleran

**Affiliations:** ^1^ College of Veterinary Medicine North Carolina State University Raleigh North Carolina USA; ^2^ Population Health & Pathobiology North Carolina State University Raleigh North Carolina USA

**Keywords:** caprine, kidney, renal calculi, renal/urinary tract, small ruminant

## Abstract

**Background:**

Symmetric dimethylarginine (SDMA), a sensitive biomarker for detecting renal injury, has not been characterized in goats. Obstructive urolithiasis (OU) is the most common urinary tract disease in male small ruminants.

**Hypothesis/Objective:**

Establish an SDMA reference interval (RI) in healthy adult goats and describe SDMA concentrations in goats with OU. We hypothesize that the SDMA RI in healthy adult goats will be similar to that of other adult veterinary species and that SDMA can be utilized to assess the renal function of goats experiencing OU.

**Animals:**

Fifty‐five healthy adult male and female goats from a university herd were enrolled for SDMA RI development. Twenty male and female goats from a university herd were enrolled for validation of the SDMA RI established. Thirteen male goats diagnosed with OU were enrolled.

**Methods:**

Clinical trial. Serum samples for all animals enrolled were collected and analyzed for SDMA using an immunoassay (IDEXX Laboratories, Inc); goats with OU had additional blood work analyzed (PCV, total solids, and serum biochemistry). Symmetric dimethylarginine and other values in goats with OU were analyzed and compared at specific time points.

**Results:**

The SDMA RI for healthy, adult goats is 8.03 μg/dL (90% CI 4.81‐11.04) to 25.93 μg/dL (90% CI 22.88‐28.97). There was no correlation identified between serum creatinine and SDMA in goats with OU.

**Conclusions and Clinical Importance:**

The SDMA RI for adult goats is higher than in other adult large animal species. Use of SDMA in goats with OU is not useful in assessing their renal function.

**Abbreviations**
[]: concentration
ASVCP: American Society for Veterinary Clinical Pathology
BUN: blood urea nitrogen
CLSI: Clinical Laboratory and Standards Institute
Cr: creatinine
GFR: glomerular filtration rate
K: potassium
LC‐MS: liquid chromatography‐mass spectrometry
Mg: magnesium
NC: North Carolina
OU: obstructive urolithiasis
RI: reference interval
*s*
: serum
SDMA: symmetric dimethylarginine
SRCC: Spearman's rank correlation coefficient
UO: urethral obstruction

## 1 INTRODUCTION

Urolithiasis is the most common cause of urinary tract disease in small ruminants.^1^ Diagnosis of obstructive urolithiasis (OU) is based on physical exam findings, advanced diagnostic imaging (eg, urinary tract‐focused ultrasonography, radiography, or both), and assessment of blood analyte derangements (eg, increases in blood urea nitrogen [BUN], creatinine [Cr], and potassium [K]). Depending on the chronicity of OU, a commonly identified blood analyte abnormality includes postrenal azotemia, where both Cr and BUN increase because of impaired renal clearance.^2^ Obstructive urolithiasis is painful and life‐threatening, and treatment often requires surgical intervention for survival.^3^ Surgical management to correct OU can be financially limiting for clients, and discerning pre‐ and postrenal azotemia from renal azotemia is difficult in these goats.^1^ Understanding a goat's renal function status before surgical treatment could be an important factor in a client's and veterinarian's decision to pursue surgical treatment of OU.

The gold standard for assessing kidney excretory function is the measurement of glomerular filtration rate (GFR).^4^ However, this technique requires the administration of an insoluble marker and the collection of timed samples.^4^ Serum (*s*) Cr is a more convenient measurement and can be used as an estimate of renal clearance, as *s*Cr is usually increased when GFR declines.^2^ Although *s*Cr is a convenient sample, interpretation requires caution as the analyte can be influenced by muscle mass and also requires approximately 60% to 75% loss of renal function to be abnormal.^5^, ^6^ Symmetric dimethylarginine (SDMA) is a biomarker correlated with GFR that allows for earlier detection than *s*Cr of decreasing renal function.^7^ In contrast to *s*Cr, SDMA is not affected by lean body mass in cats or dogs.^7^, ^8^ Therefore, SDMA is considered a more sensitive biomarker for detecting underlying kidney dysfunction than *s*Cr.^7^, ^9^ The reference range of SDMA has been determined in a few species but not in goats. In healthy dogs, cats, and adult draft horses, the upper limit is 14 μg/dL.^7^, ^10^ Serum SDMA has the potential to be utilized as a diagnostic tool in assessing renal function of goats with OU before undergoing surgery, thus helping guide client's and veterinarian's treatment decision.

We aim to establish an SDMA reference interval (RI) in healthy, adult goats and describe SDMA in goats with OU. We hypothesize that the RI for SDMA in healthy adult goats will be similar to that of other adult species. Lastly, we aim to describe SDMA concentrations in goats with OU and compare these values to Cr, BUN, Mg, or K at various time points to determine whether SDMA can be used to assess the renal function of goats with OU before surgery.

## 2 MATERIALS AND METHODS

### 2.1 Animal use and study enrollment

The NC State University Animal Care and Use Committee reviewed and approved the procedures and animal use for this study. Owner consent was obtained before enrollment of client‐owned animals.

### 2.2 Animal selection—SDMA RI development

For RI development, a minimum of 39 animals are required to yield a 95% confidence interval.^11^ With that goal, 59 apparently healthy adult male and female goats, were enrolled in March 2022 to establish an SDMA RI. These goats were from either client‐owned herds or the teaching animal units associated with NC State University. The animals were visually examined at the time of sample collection by a veterinarian. An animal was excluded if they were less than 6 months of age, or if they displayed visual signs of systemic disease (such as poor body condition, cough, nasal discharge, or diarrhea).

### 2.3 Animal selection—SDMA RI validation

Following the American Society for Veterinary Clinical Pathology (ASVCP) and Clinical Laboratory and Standards Institute (CLSI) guidelines for validating a developed RI, 20 goats different from those used to create the RI were utilized.^11^ The validation goats were adults (male and female) from a university teaching herd in Raleigh, NC. An animal was excluded if they exhibited visual signs of systemic disease using the same variables as listed above.

### 2.4 Goats with OU

A power calculation was performed to determine the number of goats needed to understand SDMA's utility as a diagnostic biomarker to indicate renal impairment in goats with OU. Considering an alpha of .05 and a power of .8, 11 goats were needed per treatment group to determine if there was a statistically significant difference. For this study, inclusion criteria included: admission to NC State University's Farm Animal Service, a clinical diagnosis of OU, and treatment for OU (surgery). Goats were diagnosed with OU based on physical examination findings, ultrasonographic findings (enlarged urinary bladder), and clinicopathologic abnormalities. After diagnosis of OU and treatment plan development, goats with client consent were enrolled in this study.

### 2.5 Blood sampling and laboratory evaluation

Blood samples for SDMA RI development and validation were collected via jugular venipuncture. Blood was collected into a syringe (3 mL) and transferred to a preservative‐free blood tube within 10 seconds of collection. The blood was allowed to clot and was then spun in a centrifuge for 10 minutes at 3000×*g*. The serum was separated within 1 minute after centrifugation into a microcentrifuge tube and stored at −20°C until processing for SDMA analysis. SDMA concentration was determined at IDEXX laboratories using an immunoassay (Memphis, Tennessee, USA).

Goats with OU had blood collected at 4 time points: presentation (0 hour), 24‐, 48‐, and 72‐hours after presentation. Blood samples were collected via jugular venipuncture at presentation and through an indwelling jugular vein catheter throughout hospitalization. Time points were chosen as these were when samples were drawn as part of the goat's initial assessment and hospitalization monitoring plan after undergoing surgery for OU. Three milliliters of blood were drawn at each time point; half of the sample was transferred to an EDTA tube, and the other half was transferred to a preservative‐free blood tube within 10 seconds of collection. The EDTA tube was inverted slowly 3 times. To perform a PCV and plasma total solids (TS), 2 nonheparinized microhematocrit capillary tubes (JorVet, Loveland, Colorado) were each filled half‐way (approximately 35 μL) with blood from the inverted EDTA tube sample and 1 end of each tube was sealed with a clay sealant. The microhematocrit capillary tubes were centrifuged for 7 minutes at 12 000×*g*. Packed cell volume was measured using a microhematocrit capillary tube reader card and plasma TS were determined using a hand‐held refractometer (CTL‐REFM‐PRSG, LW Scientific, Lawrenceville, Georgia). The sample in the preservative‐free blood tube was allowed to clot, centrifuged for 10 minutes at 3000×*g*, and the serum was separated. A full biochemistry analysis was then performed at NC State College of Veterinary Medicine's Clinical Pathology and Immunology laboratory. The remaining serum sample was transferred to a microcentrifuge tube and stored at −20°C until processing for SDMA at IDEXX laboratories (Memphis, Tennessee, USA). The biochemistry analytes of creatinine (determined using Jaffe's kinetic method), BUN, magnesium, and potassium were determined using the Cobas c501 module analyzer (Roche Diagnostics, USA) with dedicated reagents. These analytes were evaluated to understand if a correlation existed between them and SDMA at the 4 sampling time points. These analytes were chosen as they would be expected to be affected in goats with OU.

### 2.6 Statistical analysis

To determine the distribution of the data, a histogram and Shapiro‐Wilk test for normality was performed (R version 4.1.3 [2022‐03‐10]). The data were nonnormally distributed. Following CLSI guidelines, a bootstrap method (robust method) was utilized to determine a 90% CI.^11^ Using MedCalc (Version 20.305, MedCalc Software), the SDMA RI was established with the following options selected: 90% CI, double‐sided. The Reed test was used to determine if outliers were present.

A Kruskal‐Wallis test was used to determine if there was a statistically significant difference in SDMA values at different time points in goats with OU. The Spearman's rank correlation coefficient (SRCC) was used to determine if serum creatinine, BUN, magnesium, or potassium were correlated with SDMA at the 4 sampling time points (R version 4.1.3 [2022‐03‐10]). A coefficient value close to 1 indicates a strong, positive correlation. A *P* < .05 was considered statistically significant.

## 3 RESULTS

### 3.1 SDMA RI development

**FIGURE 1:**
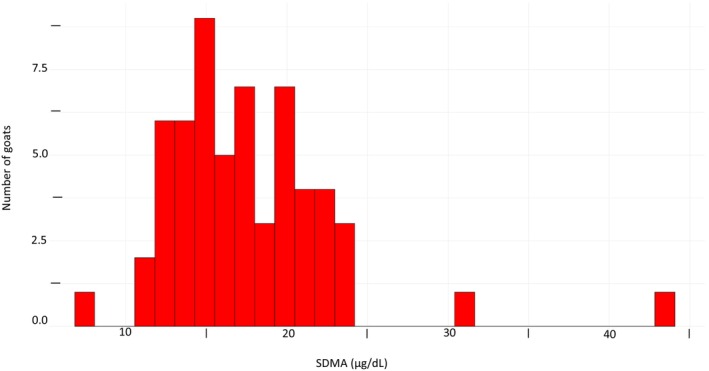
Histogram of (n = 59) symmetric dimethylarginine (SDMA) results (μg/dL) for control goats. Five intact males and 54 female goats were enrolled. Males were Boers (≥6 months old, approximately 1 year old); females were Boer crosses (n = 19, ≥6 months old; exact ages unknown) and lactating Alpine does (n = 35, 1st to 6th lactation). All animals were apparently healthy (health status determined by visual exam). The histogram depicts a nonnormally distributed study cohort and a right‐skewed distribution.

Five intact males and 54 female goats were enrolled to establish an SDMA RI (n = 59). Males were Boers (≥6 months old, approximately 1 year old); females were Boer crosses (n = 19, ≥6 months old; exact ages unknown) and lactating Alpine does (n = 35, 1st to 6th lactation). The samples were collected in March 2022. Figure 1 depicts a histogram of the SDMA control sample data, with the results being nonnormally distributed and right‐skewed. The resulting SDMA RI was 8.03 μg/dL (90% CI 4.81‐11.04) at the lower end to 25.93 μg/dL (90% CI 22.88‐28.97) at the higher end.

### 3.2 SDMA RI validation

To validate the SDMA RI established (8.03 μg/dL [90% CI 4.81‐11.04] to 25.93 μg/dL [90% CI 22.88‐28.97]), 4 male and 16 female goats were enrolled (n = 20) in July 2023. Ten goats were Kiko and 10 were Boer. The age range was 8 months to 6 years old (median = 2 years). The descriptive statistics for the SDMA validation study cohort are as follows—mean: 15.5 μg/dL; median: 14 μg/dL; range: 10 μg/dL to 37 μg/dL; mode: 13 μg/dL. Table S1 includes the RI validation study cohort demographics and results.

### 3.3 Goats with OU

Twelve wethers and 1 buck were enrolled as goats with OU (n = 13) from August 2022 to June 2023. The breeds represented were: Mixed (n = 5), Pygmy (n = 3), Myotonic (n = 2), Nigerian Dwarf (n = 1), Nubian (n = 1), and French Alpine (n = 1). The age range was 9 months to 4 years old (median = 2 years). The age data were nonnormally distributed as determined by the Shapiro‐Wilk test (*P* = 3.5 × 10^−9^). Goats received ancillary treatment for OU including IV fluid support (0.9% sodium chloride, Normosol‐R, or lactated ringers solution), antimicrobial therapy (ceftiofur sodium—50 mg/mL, 2.2 mg/kg SC q24h or ampicillin trihydrate—400 mg/mL, 11 mg/kg IM q24h), an analgesic agent (phenazopyridine 95 mg tablets, 4 mg/kg PO q24h), and nonsteroidal anti‐inflammatory therapy (flunixin meglumine—50 mg/mL, 1.1 mg/kg IV q12h or meloxicam—15 mg tablets, 1 mg/kg PO q24h). Treatment and medication selection varied among clinicians and the duration of therapy changed depending on the duration of hospitalization of each goat.

**FIGURE 2:**
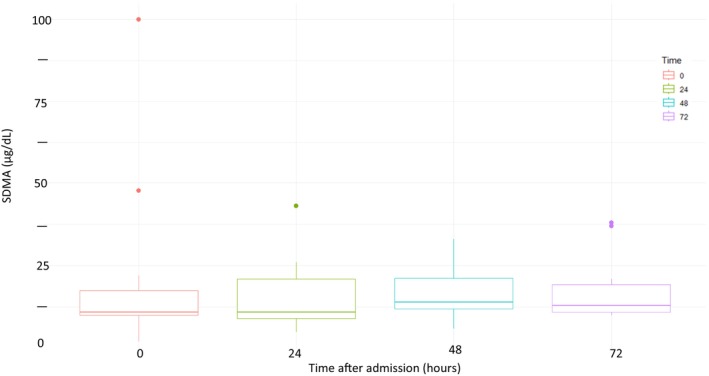
Box and whisker plot of symmetric dimethylarginine (SDMA) results (μg/dL) for goats diagnosed with clinical obstructive urolithiasis over sampling time points: 0‐, 24‐, 48‐, and 72‐hours after presentation. Thirteen male goats were enrolled (12 wethers and 1 buck). The breeds represented were: Mixed (n = 5), Pygmy (n = 3), Myotonic (n = 2), Nigerian Dwarf (n = 1), Nubian (n = 1), and French Alpine (n = 1). The age range was 9 months to 4 years old (median = 2 years). No statistical significance between time points was identified.

To determine if a statistically significant difference existed among the SDMA results at the 4‐time points sampled (0‐, 24‐, 48‐, and 72‐hours after presentation) in goats with OU, a Kruskal‐Wallis test was used. No statistical difference was found (*P* = .63). Figure 2 depicts the SDMA results for goats diagnosed with OU, over the study sampling points (0, 24, 48, and 72 hours); there was no statistical significance identified between time points. The SRCC was used to determine if serum creatinine, BUN, magnesium, or potassium have any correlation to SDMA at the 4 sampling time points of the study. There was no statistically significant correlation of *s*Cr to SDMA or *s*Mg and SDMA at the 4 sampling time points (*s*Cr *P*‐values: 0 hour = .17, 24 hours = .44, 48 hours = .36, and 72 hours = .63; *s*Mg *P*‐values: 0 hour = .10, 24 hours = .26, 48 hours = .11, and 72 hours = .23). Similarly, there was no correlation between BUN and SDMA at 24‐ (*P* = .07) and 48‐hours (*P* = .07) after presentation and no association between *s*K and SDMA at 24‐ (*P* = .24), 48‐ (*P* = .57), and 72‐hours (*P* = .21) after presentation. There was, however, a statistically significant and moderate positive correlation between BUN and SDMA at 0 hours (*r* = .69; *P* = .01) and strong positive correlation at 72 hours (*r* = .78; *P* = .01). Additionally, there was a statistically significant, moderate positive association between *s*K and SDMA at time 0 hour (*r* = .67; *P* = .01). Table S2 includes SDMA, *s*Cr, BUN, *s*Mg, *s*K, PCV, and TS for the goats with OU at 0, 24, 48, 72 hours. Table S3 includes descriptive statistics of the SDMA, *s*Cr, BUN, *s*Mg, and *s*K at 0, 24, 48, and 72 hours for the goats with OU.

## 4 DISCUSSION

Our study establishes the adult goat SDMA RI as 8.03 μg/dL (90% CI 4.81‐11.04) to 25.93 μg/dL (90% CI 22.88‐28.97). We determined a positive correlation between *s*K and SDMA and between BUN and SDMA at time 0 hour. However, neither of the positive correlations aids in discerning renal function of a goat with OU before undergoing surgery for OU. No correlation between *s*Cr and SDMA was identified. Therefore, at this time SDMA cannot be used as a diagnostic marker in determining whether goats with OU have appropriate renal function before undergoing surgery.

Within our study, only 1 of the validation goat's SDMA concentration was outside of the RI established (Goat #17—8 mo Kiko male, SDMA: 37 μg/dL; RI: 8.03 μg/dL [90% CI 4.81‐11.04] to 25.93 μg/dL [90% CI 22.88‐28.97]). The RI established in this study is valid as no more than 2 results were outside proposed RI.^11^ Regardless, it is important to note that the upper limit of SDMA in adult goats is higher than those reported in adult cats, dogs, and horses.^7^, ^9^, ^10^ The SDMA reference range in neonatal foals (≤30 days old), puppies, and kittens is higher than in adults.^12^, ^13^ The goats sampled for the RI and SDMA validation sampling were older than 6 months of age, but the exact ages of the RI animals were unknown. Although OU can occur at any age in goats, animals less than 6 months of age were excluded for establishing the RI as differences in laboratory interpretation of other biochemistry analytes were previously identified in goat kids (<4 months old) and in juvenile goats (aged >4 but <6 months).^3^, ^14^, ^15^ Age might have contributed to a higher RI as age was the key component identified affecting differences in clinical chemistry variables and interpretation in goats.^15^ However, there are no studies evaluating SDMA concentration in different age groups of goats to clarify if a period of increased SDMA concentration should be expected within the 1st year of life. Currently, the higher SDMA concentration in adult goats observed in this study is not fully understood.

In our study, a strong, positive correlation between *s*K and SDMA was found at presentation (time 0 hour) in goats experiencing OU. Potassium concentration ([K]) is mainly regulated by 2 processes—1 of which is renal excretion.^2^ Subsequently, hyperkalemia can occur in cases of urinary obstruction.^2^ Clinically, evaluating a goat's [K] is crucial, as derangements (both above or below the reference range) in this analyte can cause cardiac abnormalities and lead to death. The positive correlation identified between *s*K and SDMA indicates the severity of renal compromise secondary to urinary obstruction and blockage. However, the correlation does not yield any information to the state of the goat's renal function, therefore the correlation cannot be utilized to determine the prognosis of a goat with OU.

Although there was a positive correlation between BUN and SDMA at presentation (time 0 hour) and at 72 hours, BUN alone is not enough to clinically assess a ruminant's renal function impairment. In ruminants, BUN is less sensitive to decreases in GFR than *s*Cr, as urea is excreted in saliva and metabolized by bacteria in the rumen.^2^, ^5^ In our study, no correlation was identified between *s*Cr and SDMA or *s*Mg and SDMA at the 4 sampling points. A study performed in cats with urethral obstruction (UO) found a positive association between SDMA and *s*Cr at presentation and 24 hours, but not at 5 to 20 days after decompression.^16^ This study concluded the positive association identified between SDMA and *s*Cr was indicative of postrenal azotemia, but not intrinsic renal disease in cats with UO. Similarly, we found that SDMA did not serve as an indicator of renal function in a goat with OU. Within veterinary medicine, the application of SDMA has been for identifying earlier detection of impaired renal function (when compared to *s*Cr), as SDMA has an inverse relationship with GFR.^7^, ^17^ In OU, GFR decreases because of an increase in nephron tubular pressure, an effect that is largely corrected once the obstruction has been resolved.^16^ Therefore, although SDMA correctly identifies a change in GFR in goats with OU, these changes are often not permanent because of the disease process of OU (which is inherently different from chronic kidney disease for example). Thus, similar to cats, SDMA does not serve as a tool for assessing the true renal function of goats with OU.^16^

Although a sample size calculation was performed before conducting this study, a possible explanation for failing to identify a correlation between SDMA and *s*Cr could be the number of goats with OU enrolled (n = 13). A correlation was identified in a cat study, in which 25 cats with UO were enrolled.^16^ Similarly, in a horse study, 165 horses were enrolled, and a correlation was identified between SDMA and *s*Cr.^10^ Ultimately, understanding the limitations of SDMA in goats with OU is important as SDMA alone would not aid a clinician or a client in understanding the renal function and therefore prognosis of a goat experiencing OU before undergoing surgery to relieve the obstruction.

Our study group represented goats in a diseased state (OU). Studies evaluating the correlation between SDMA and Cr in healthy small and large animals exist, but limited studies are available evaluating these variables in a diseased state.^9^, ^10^ SDMA is not a beneficial biomarker to categorize critical illness in dogs.^18^ Similarly, in horses there is not enough evidence to indicate that SDMA is a better biomarker of kidney dysfunction than other methods in horses with acute kidney injury.^9^

There were limitations to our study. Our RI and validation study cohort had an overrepresentation of Boer, Boer crosses, and Alpine breeds. This is in contrast with the mixed group of breeds of goats with OU in the study. However, in dogs and cats, SDMA is not affected by muscle mass or breed, and in adult horses, the clinical difference found between SDMA in draft breeds was not clinically relevant.^10^, ^19^ One of the primary objectives of our study was to establish an SDMA RI for adult goats and apply this RI to goats experiencing OU. Our RI group displayed an overrepresentation of females whereas OU cases are almost exclusively males. To date, a sex dimorphism in SDMA concentration has not been identified in dogs or horses.^9^, ^10^, ^20^, ^21^ In cats, however, SDMA concentration is higher in overweight male cats.^22^ Therefore, although possible, it currently seems unlikely that a sex dimorphism in SDMA concentration would exist in goats. Thus, the overrepresentation of females with the RI established should have little to no effect in assessing the SDMA concentrations obtained for the OU cohort.

Second, blood samples for RI establishment, goats with OU, and RI validation were not run in duplicate. Additionally, only the SDMA IDEXX immunoassay was used instead of comparing the results to the gold standard of liquid chromatography‐mass spectrometry (LC‐MS). However, the IDEXX SDMA immunoassay has a strong correlation to samples run with LC‐MS in large animals.^10^ Furthermore, this immunoassay is a test that is clinically available and easily accessible. As a result, the samples in this study were only run using the immunoassay.

A blood sample at the time of foley tube removal (typically 10‐14 days after tube cystostomy surgery) could have been performed to further characterize changes in SDMA throughout hospitalization; however, there were no differences in mean *s*Cr or SDMA concentration in cats with UO at 24 hours to 5 to 20 days after decompression.^16^ In the current study, goats were not routinely reevaluated by NC State's Farm Animal Service after foley tube removal and discharge thus limiting access to follow‐up samples. With the emergency nature of goats with OU, the exact duration of time that a goat had been obstructed was unknown. Therefore, it was not possible to incorporate this variable into the analysis or draw conclusions on whether the length of time a goat was obstructed would affect the SDMA results obtained from goats with OU. In critically ill humans, SDMA is significantly higher compared to healthy controls; however, a preliminary study in dogs did not find SDMA to be a prognostic indicator for survival.^19^ Urine was also not collected during this study.

In conclusion, SDMA values in healthy goats are higher than other adult large animal species, but the effect of breed, age, and disease state on SDMA values in goats should be further evaluated.

## CONFLICT OF INTEREST DECLARATION

Authors declare no conflict of interest.

## OFF‐LABEL ANTIMICROBIAL DECLARATION

Goats with OU received ceftiofur sodium 2.2 mg/kg (50 mg/mL) SC every 24 hours (the duration of treatment varied for each goat) and phenazopyridine 4 mg/kg (95 mg) PO once every 12 hours while in hospital. Submissions for extended extra label drug use were submitted to the Food Animal Avoidance Databank (FARAD). FARAD stated for ceftiofur sodium at a dose of 2.2 mg/kg administered SC for the longest administration (56 days) had an associated meat withdrawal interval of 17 days. Because there are no current data regarding phenazopyridine, a conservative meat withdrawal interval of 30 days was used for multiple doses. One goat was administered ampicillin trihydrate at a dose of 11 mg/kg IM once a day for 4 days; this has an associated meat withdrawal interval of 10 days.

## INSTITUTIONAL ANIMAL CARE AND USE COMMITTEE (IACUC) OR OTHER APPROVAL DECLARATION

Approved by North Carlina State University IACUC, #22‐151, and North Carlina State University Hospital board, approval date August 2022.

## HUMAN ETHICS APPROVAL DECLARATION

Authors declare human ethics approval was not needed for this study.

## Supporting information


**Table S1.** Symmetric dimethylarginine (SDMA) reference interval (RI) validation study cohort demographics and respective SDMA (μg/dL) results.
**Table S2.** Clinical data results of male goats with obstructive urolithiasis at 4 sampling time points. Symmetric dimethylarginine (SDMA), creatinine (Cr), blood urea nitrogen (BUN), magnesium (Mg), potassium (K), packed cell volume (PCV), and total solids (TS).
**Table S3.** Descriptive statistics of obstructive urolithiasis study cohort at 4 sampling time points for symmetric dimethylarginine (SDMA), creatinine (Cr), blood urea nitrogen (BUN), magnesium (Mg), and potassium (K). Data are shown as median (quartile 1, quartile 3).
