# Supplementary material for: Validation of a reference interval for symmetric dimethylarginine in healthy goats and its comparison to values in goats with obstructive urolithiasis
Source: J Vet Intern Med. 2024 Aug 17;38(5):2807–13. doi: 10.1111/jvim.17162 (PMC11423465; doi:10.1111/jvim.17162)
Supplement: Supplementary file 1 — Table S1. Symmetric dimethylarginine (SDMA) reference interval (RI) validation study cohort demographics and respective SDMA (μg/dL) results. Table S2. Clinical data results of male goats with obstructive urolithiasis at 4 sampling time points. Symmetric dimethylarginine (SDMA), creatinine (Cr), blood urea nitrogen (BUN), magnesium (Mg), potassium (K), packed cell volume (PCV), and total solids (TS). Table S3. Descriptive statistics of obstructive urolithiasis study cohort at 4 sampling time points for symmetric dimethylarginine (SDMA), creatinine (Cr), blood urea nitrogen (BUN), magnesium (Mg), and potassium (K). Data are shown as median (quartile 1, quartile 3). [file JVIM-38-2807-s001.docx]

**Table S1.** Symmetric dimethylarginine (SDMA) reference interval (RI) validation study cohort demographics and respective SDMA (ug/dL) results.

**Table S2.** Clinical data results of male goats with obstructive urolithiasis at four sampling time points. Symmetric Dimethylarginine (SDMA), Creatinine (Cr), Blood urea nitrogen (BUN), Magnesium (Mg), Potassium (K), Packed cell volume (PCV), Total solids (TS).

**Table S3.** Descriptive statistics of obstructive urolithiasis study cohort at four sampling time points for Symmetric dimethylarginine (SDMA), Creatinine (Cr), Blood urea nitrogen (BUN), Magnesium (Mg), and Potassium (K). Data are shown as median (quartile 1, quartile 3).
